# Self-Reported Vaccination Coverage and Occupational Immunization Gaps Among Veterinarians in Málaga, Spain: A One Health Perspective

**DOI:** 10.3390/vetsci13080739

**Published:** 2026-07-25

**Authors:** Rosa López-Gigosos, Eduardo Martínez-Manzanares, Eloísa Mariscal-López, Fernando Fariñas-Guerrero, Juan A. de Luque-Ibañez, Jose L. Peñate-García, Antonio J. Villatoro

**Affiliations:** 1 Department of Public Health and Psychiatry, Faculty of Medicine, University of Malaga (UMA), Boulevard Louis Pasteur, 32, 20010 Málaga, Spain; eml@uma.es; 2 Malaga Biomedical Research Institute and Nanomedicine Platform (IBIMA BIONAND Platform), Avenida Severo Ochoa, 35, 29590 Málaga, Spain; emmanzanares@uma.es; 3 Standing Committee of the One Health Chair, Málaga University and Official College of Veterinarians, 29010 Málaga, Spain; farinas.inmunologia@gmail.com (F.F.-G.); deluqueibanez@gmail.com (J.A.d.L.-I.); jpenategarcia@gmail.com (J.L.P.-G.); ajvillatoro@immunestem.com (A.J.V.); 4 Department of Microbiology, University of Malaga, Avda. Cervantes, 2, 29071 Málaga, Spain; 5 Institute of Clinical Immunology and Infectious Diseases, 29010 Málaga, Spain; 6 Official College of Veterinarians of Málaga, Passage Esperanto, 1, 29007 Málaga, Spain; 7 Immune Stem (Immunology and Cell Therapy), C/Miraflores del Palo, 14, 29018 Málaga, Spain

**Keywords:** veterinarians, vaccination coverage, occupational immunization, zoonoses, occupational health, One Health

## Abstract

Veterinarians are routinely exposed to occupational infectious hazards at the human–animal interface, making vaccination an important preventive measure within a One Health framework. However, evidence on vaccination coverage and immunization practices in this professional group remains limited. This study assessed self-reported vaccination coverage, attitudes toward vaccination, perceived barriers, and training needs among registered veterinarians in Málaga, Spain. A cross-sectional anonymous survey was distributed to all actively practicing veterinarians registered with the Official Veterinary Association of Málaga. The questionnaire collected sociodemographic and professional information, self-reported vaccination status, attitudes toward vaccination, and perceived training needs. A total of 164 veterinarians participated (response rate: 15.2%). Given the voluntary nature of participation, findings should be interpreted as descriptive. Vaccination coverage was low for several vaccines relevant to occupational or adult immunization, including seasonal influenza (21.3%), rabies (23.2%), and tetanus–diphtheria–pertussis boosters (47.3%). Nearly half of respondents (49.1%) were uncertain about their hepatitis B vaccination status. In contrast, COVID-19 vaccination uptake was high (96.9% received at least one dose). Although most participants considered vaccination highly important, self-reported coverage remained limited for several recommended vaccines. Qualitative responses identified barriers related to access to vaccination, information, and the absence of structured occupational vaccination programmes. Among the surveyed veterinarians in Málaga, these findings support the need for clearer occupational vaccination recommendations, improved access to immunization services, and enhanced vaccinology training. Such measures could strengthen occupational health protection and support One Health prevention strategies at the human–animal interface.

## Simple Summary

Veterinarians are regularly exposed to animals and zoonotic pathogens, making vaccination an important component of occupational health protection. However, information on vaccination coverage among veterinary professionals remains limited in many countries, including Spain. This cross-sectional survey assessed self-reported vaccination coverage, attitudes toward vaccination, perceived barriers, and training needs among registered veterinarians in Málaga, Spain. Although participants reported highly positive attitudes toward vaccination, coverage for some recommended vaccines remained suboptimal, while nearly half of participants were uncertain about their hepatitis B vaccination status. Qualitative responses highlighted barriers related to access to vaccines, insufficient information, and the absence of structured occupational vaccination programmes. These findings suggest that improving vaccine uptake among veterinarians requires not only greater awareness but also easier access to recommended vaccines, evidence-based occupational vaccination programmes, and stronger institutional support. Strengthening vaccination among veterinary professionals may contribute to occupational health protection and reinforce preparedness for zoonotic diseases within a One Health framework.

## 1. Introduction

Veterinary professionals work at the human–animal–environment interface and are routinely exposed to biological hazards through direct contact with animals, animal products, and contaminated environments. Compared with the general population, veterinarians have an increased occupational risk of several infectious diseases, including zoonotic infections such as rabies, avian influenza, and Q fever, with higher seroprevalence reported for some occupationally acquired pathogens in specific practice settings [1]. Vaccination therefore represents an important component of occupational health protection and a key preventive strategy within the One Health framework [2].

The vaccination needs of veterinary professionals encompass different categories of vaccines. Some are recommended because of occupational exposure (e.g., rabies and seasonal influenza in professionals at risk of exposure to animal influenza viruses), whereas others correspond to routine adult immunization (e.g., tetanus–diphtheria–pertussis boosters) or protection against blood-borne occupational hazards (e.g., hepatitis B). Additional vaccines, such as hepatitis A or yellow fever, may be indicated according to travel or individual risk assessment. Appropriate vaccination should therefore be guided by both routine immunization schedules and profession-specific occupational risk [3,4,5].

Despite these recommendations, few countries have developed comprehensive and harmonized vaccination policies specifically for veterinary professionals, resulting in heterogeneous guidance and variable implementation [6,7]. Vaccination of veterinarians is supported by occupational, public health, and One Health considerations, including protection of workers, reduction in disease transmission, maintenance of workforce capacity, and mitigation of economic impact [8,9]. Professional organizations have consequently recommended vaccination against influenza, COVID-19, hepatitis A and B, rabies, and tetanus–diphtheria–pertussis where appropriate [10,11,12]. Nevertheless, internationally harmonized recommendations specifically tailored to veterinary professionals remain limited, highlighting the need for coordinated guidance from veterinary and One Health organizations.

In Spain, dissemination and implementation of occupational vaccination recommendations among veterinarians remain limited, and empirical data on vaccination coverage, attitudes, and barriers are scarce. This evidence gap hampers the design of effective occupational immunization strategies and limits the development of evidence-based recommendations for veterinary professionals.

The present study aimed to assess self-reported vaccination coverage, attitudes toward vaccination, perceived barriers, and training needs among registered veterinarians in the province of Málaga, Spain. By providing empirical evidence from an underrepresented occupational group, this study seeks to inform the development of evidence-based vaccination policies and occupational immunization programmes, improve access to recommended vaccines, and support harmonized vaccination recommendations for veterinary professionals at national and international levels.

## 2. Materials and Methods

### 2.1. Study Design

A cross-sectional descriptive study was conducted using a voluntary, anonymous questionnaire administered between May and June 2025. The survey (Appendix A) was available in both electronic and paper formats, allowing participants to choose their preferred mode of completion. The study was designed to describe self-reported vaccination coverage, attitudes toward vaccination, and qualitative perceptions regarding occupational vaccination among practicing veterinarians.

### 2.2. Study Population and Setting

The study population comprised actively practicing veterinarians registered with the Official Veterinary Association of Málaga Province (Ilustre Colegio Oficial de Veterinarios de Málaga), Spain. At the time of the study, the professional register included 1119 licensed veterinarians, of whom 39 were retired and were therefore excluded from the survey distribution. A small number of registered members resided or practiced outside Spain but remained professionally registered in Málaga. Consequently, the survey was directed exclusively to veterinarians who were actively practicing at the time of the study, representing a wide range of professional activities, including companion animal practice, livestock medicine, public health, food safety, and other veterinary fields.

### 2.3. Recruitment and Sample

The questionnaire was distributed to all actively practicing veterinarians registered with the Official Veterinary Association of Málaga through institutional communication channels. An electronic version was disseminated by email, and paper copies were made available through the professional association. Participation was voluntary and anonymous, and no incentives were offered.

To minimize duplicate participation, respondents were asked to complete the questionnaire only once, regardless of the administration format. Paper questionnaires were collected by the professional association, and duplicate submissions were checked during data management; no duplicate responses were identified.

Because participation was voluntary, no formal sample size calculation was performed. The resulting sample should therefore be considered a convenience sample, and findings are interpreted descriptively rather than as population estimates.

### 2.4. Survey Instrument

An ad hoc questionnaire was specifically developed for this study (Apendix A). Its content was informed by national and international recommendations on vaccination of healthcare and veterinary professionals, published occupational immunization guidance, and the research objectives of the study [2,6,7,10]. The questionnaire was designed to collect descriptive information on vaccination coverage and factors potentially influencing vaccine uptake among veterinarians.

Before implementation, the questionnaire underwent face-validity assessment through pilot testing with 20 actively practicing veterinarians who were not included in the final study sample. Based on participants’ feedback, only one modification was introduced: the original open-ended question on the perceived importance of vaccination was replaced by a numerical rating scale ranging from 1 (lowest importance) to 10 (highest importance) to facilitate standardized responses and quantitative analysis. All pilot participants reported that the questionnaire was clear, understandable, and easy to complete, and no further modifications were considered necessary.

The final questionnaire included the following sections:

Sociodemographic characteristics (age and sex);

Professional characteristics (work setting and main professional activity);

Self-reported vaccination status for selected vaccines relevant to adult immunization and veterinary occupational practice, including seasonal influenza, COVID-19, hepatitis B, tetanus–diphtheria–pertussis (Td/Tdap), rabies, and travel-related vaccines;

Perceived importance of vaccination, assessed using a numerical rating scale from 1 to 10;

An open-ended question inviting participants to provide additional comments or suggestions regarding vaccination.

Themes related to perceived barriers to vaccination, training needs, and recommendations for improving occupational vaccination programmes were identified inductively through thematic analysis of the responses to this open-ended question.

Hepatitis A vaccination was not included as an independent item because it is not routinely recommended in the Spanish adult immunization schedule. Instead, it was considered within the section on travel-related vaccination, reflecting current national vaccination recommendations.

Completion of the questionnaire required approximately 10–15 min.

### 2.5. Data Collection Period

Data collection was conducted between May and June 2025. The electronic questionnaire was distributed through the institutional communication channels of the Official Veterinary Association of Málaga, while paper questionnaires were made available at the Association headquarters for veterinarians who preferred this format.

To avoid duplicate participation, respondents were instructed to complete the questionnaire only once, regardless of the administration format. During data management, responses were reviewed for potential duplicate entries, and none were identified.

Questionnaires were anonymous, and no personal identifiers allowing participant identification were collected.

### 2.6. Ethical Considerations

The study was approved by the Provincial Research Ethics Committee of Málaga (CEIm; reference SICEIA-2025-000199). The study was conducted in accordance with regional regulations (Decree 8/2020, Andalusian Official Gazette No. 24, 5 February 2020). Participation was anonymous, and informed consent was obtained implicitly through voluntary completion of the questionnaire.

The study was promoted by the One Health Chair (code OH0002025001). The protocol was finalized on 20 January 2025, with an amendment on 4 February 2025, when the informed consent document was also approved.

The study was conducted in accordance with the principles of the Declaration of Helsinki and applicable Spanish data protection legislation.

### 2.7. Data Analysis

Statistical analyses were primarily descriptive because the study was based on a voluntary convenience sample and was not designed to estimate vaccination coverage in the overall veterinary population. Continuous variables are presented as means and standard deviations (SD), whereas categorical variables are presented as frequencies and percentages. Unless otherwise specified, percentages were calculated using the number of respondents providing valid answers for each question as the denominator.

Vaccination status was self-reported and was not verified through vaccination records or serological testing. Consequently, the results should be interpreted as participants’ reported vaccination histories rather than confirmed immunization status.

Differences between survey respondents and the source population were explored descriptively according to age and sex. To evaluate the extent of potential participation bias, post-stratification weights based on the age and sex distribution of the actively practicing veterinary population registered in Málaga were calculated. These weights were used as a sensitivity analysis to assess the potential influence of differential participation. Because the primary objective of the study was descriptive and the survey was based on a voluntary convenience sample, the manuscript presents the original unweighted descriptive results throughout for consistency and ease of interpretation.

Thematic analysis was performed on responses to the open-ended question using an inductive descriptive approach. Two investigators (R.L.-G. and A.J.V.) independently reviewed and coded all responses. Codes were subsequently grouped into broader themes through discussion and consensus. A third investigator (E.M.-L.) was designated to resolve disagreements if necessary; however, no disagreements requiring arbitration occurred. Individual comments could contribute to more than one thematic category. Consequently, percentages reported for qualitative themes refer to the proportion of respondents providing comments (*n* = 47), and totals may exceed 100%.

Statistical analyses were performed using IBM SPSS Statistics version 29 (IBM Corp., Armonk, NY, USA).

## 3. Results

### 3.1. Participation and Sample Considerations

Between May and June 2025, a voluntary anonymous survey was conducted among actively practicing veterinarians registered with the Official Veterinary Association of Málaga Province, Spain. Of the 1119 veterinarians registered with the Official Veterinary Association of Málaga, 39 retired members were excluded from the survey distribution. Therefore, the eligible study population comprised 1080 actively practicing veterinarians, of whom 164 completed the questionnaire (response rate: 15.2%).

Because participation was voluntary, the study sample constituted a convenience sample rather than a representative sample of the veterinary population. Compared with the eligible registered population, respondents were slightly younger and included a higher proportion of women, indicating potential participation bias. Therefore, the findings should be interpreted as descriptive of the surveyed veterinarians rather than directly generalizable to all veterinarians registered in Málaga.

To assess the potential impact of differential participation, post-stratification weighting based on age and sex distributions of the eligible registered veterinary population was performed as a sensitivity analysis. Since weighted estimates were very similar to the original results, only the unweighted data are presented in the tables and throughout the Results section for clarity. Nevertheless, residual selection bias cannot be excluded because weighting cannot account for unmeasured factors associated with study participation.

### 3.2. Sociodemographic and Professional Characteristics

The sociodemographic and professional characteristics of the surveyed veterinarians are summarized in Table 1, including age, sex, work setting, and main professional activity.

The mean age of participants was 45.6 ± 12.8 years. Women accounted for 63.4% of respondents (*n* = 104), men for 36.0% (*n* = 59), and one participant (0.6%) did not report sex.

Women were younger on average than men (42.9 ± 11.7 vs. 50.3 ± 13.5 years). Most respondents were between 40 and 59 years of age, with women predominating in the younger age groups and men in the older age groups.

Most respondents reported working primarily in urban settings (67.5%), followed by rural settings (16.6%) and mixed urban–rural practice (2.5%). A further 9.8% were veterinarians in training or did not report their work setting.

Companion animal practice was the predominant professional activity, representing 65.9% of respondents. Other reported activities included public health (11.0%), mixed companion animal/livestock practice (5.5%), animal health services (2.4%), food and environmental safety (2.4%), and other professional roles (9.1%). Veterinarians working in companion animal practice were younger on average than those working in public health or food and environmental safety.

### 3.3. Vaccination Status

Self-reported vaccination status for selected vaccines relevant to routine adult immunization and veterinary occupational practice is summarized in Table 2. Results are presented as unweighted frequencies and percentages.

#### 3.3.1. Seasonal Influenza Vaccination

During the 2024–2025 influenza season, 21.3% of respondents reported having received seasonal influenza vaccination, whereas 78.7% reported not being vaccinated. Vaccinated participants were older on average than those who were not vaccinated (52.2 vs. 43.8 years).

Influenza vaccine uptake increased with age, from 11.5% among veterinarians younger than 40 years to 47.0% among those aged 60 years or older. Coverage was also higher among men than women, consistent with the older age distribution of male respondents.

Regarding influenza vaccination during the previous ten years, 52.4% reported never having been vaccinated, 37.8% reported vaccination in some years, and only 7.3% reported annual vaccination.

#### 3.3.2. COVID-19 Vaccination

COVID-19 vaccination coverage was high. Overall, 96.9% of respondents reported having received at least one vaccine dose during the pandemic, including 62.8% who had received three or more doses and 34.1% who had received one or two doses. Only 3.0% reported never having been vaccinated.

Vaccine uptake was slightly higher among women than men (97.7% vs. 94.8%). No relevant age differences were observed across vaccination categories.

#### 3.3.3. Hepatitis B Vaccination

Considerable uncertainty was observed regarding hepatitis B vaccination history. Nearly half of respondents (49.1%) reported not knowing whether they had previously been vaccinated. Sixteen-point six percent reported completion of the three-dose vaccination schedule, 18.4% reported previous vaccination without recalling the number of doses, and 16.0% reported never having been vaccinated.

Respondents reporting completion of the vaccination schedule were younger on average than those who reported no vaccination or were uncertain about their vaccination history.

#### 3.3.4. Tetanus, Diphtheria, and Pertussis Vaccination

Regarding tetanus–diphtheria or tetanus–diphtheria–pertussis booster vaccination during the previous ten years, 47.3% reported having received at least one booster dose, 45.4% reported no booster vaccination, and 7.4% were unsure of their vaccination history.

Women more frequently reported having received a booster dose than men. No relevant differences were observed according to work setting or professional activity.

#### 3.3.5. Rabies Vaccination

Overall, 23.2% of respondents reported previous rabies vaccination, whereas 75.0% reported never having been vaccinated and 1.2% were unsure of their vaccination history.

No relevant age differences were observed between vaccinated and unvaccinated respondents. Because professional exposure to rabies varies according to veterinary activity, vaccination status according to occupational indication is considered in the Discussion.

#### 3.3.6. Travel-Related Vaccination

Travel-related vaccination was reported by 20.1% of respondents, most commonly yellow fever, hepatitis A, and typhoid fever.

#### 3.3.7. Childhood Vaccination Status

Most respondents (69.0%) believed they had completed the childhood immunization schedule, while 23.8% reported partial coverage and 6.7% were uncertain. 

### 3.4. Perceived Importance of Vaccination

**Figure 1:**
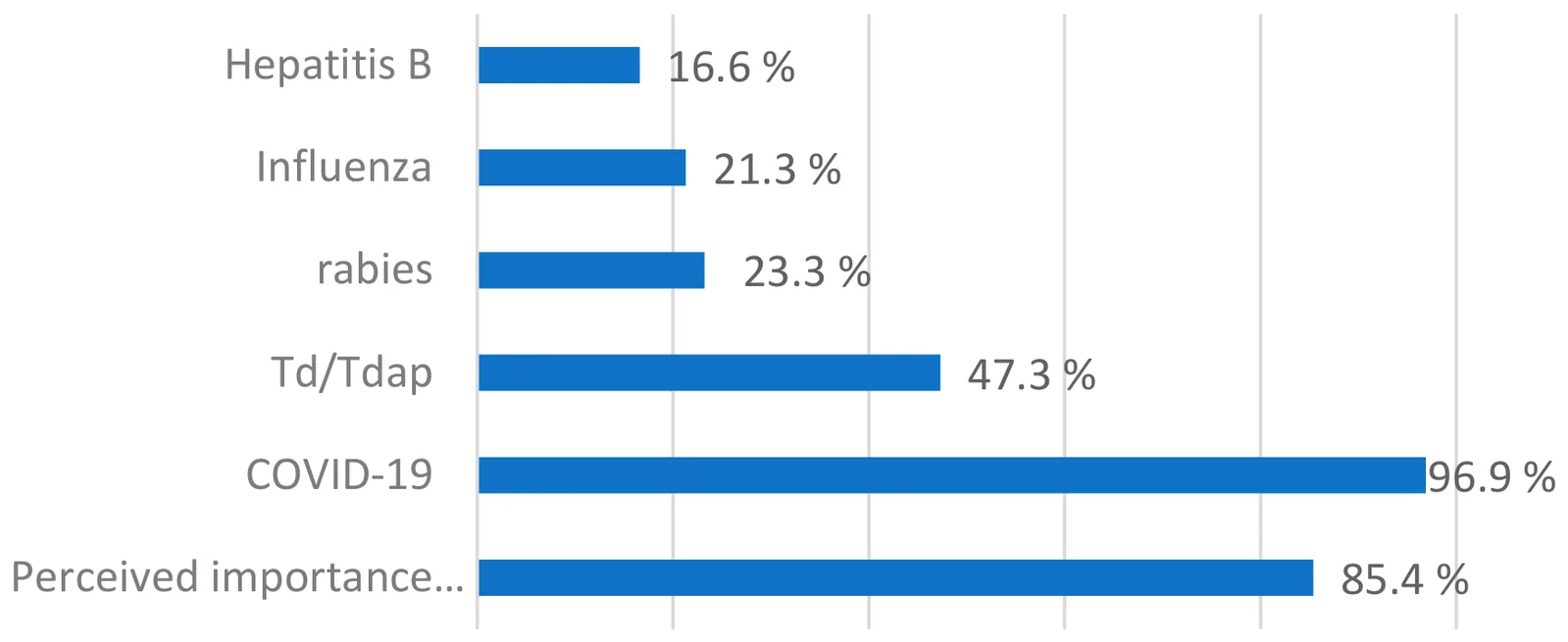
Overall perceived importance of vaccination and self-reported vaccination coverage for selected vaccines among surveyed veterinarians in Málaga, Spain (2025). General perceived importance of vaccination (participants assigning scores of 8–10 on a 10-point scale) and self-reported vaccination coverage for selected vaccines among surveyed veterinarians in Málaga, Spain (2025). Perceived importance was assessed once as an overall attitude toward vaccination and was not measured separately for individual vaccines.

Most respondents assigned high importance to vaccination as a measure of individual and public health protection. Overall, 85.4% rated its importance between 8 and 10 on a 10-point scale, with 90 respondents assigning the maximum score (Figure 1). Self-reported vaccination coverage varied considerably across vaccines, ranging from 16.6% for hepatitis B (complete three-dose schedule) to 96.9% for COVID-19 (at least one dose). Because perceived importance was assessed as a general attitude rather than separately for each vaccine, these findings should be interpreted descriptively and do not constitute a direct measure of vaccination intention for individual vaccines.

### 3.5. Qualitative Findings from Open-Ended Responses

**Figure 2:**
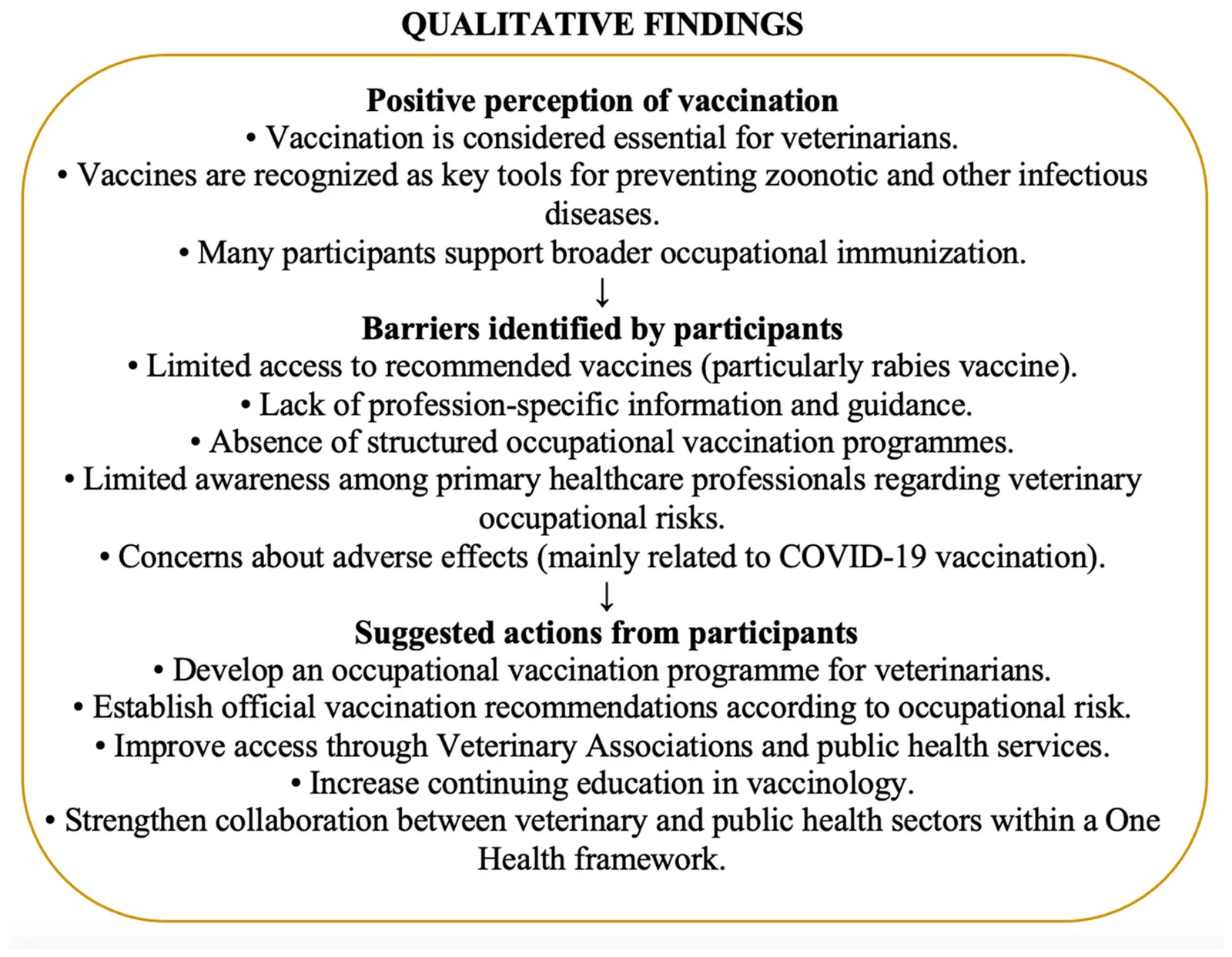
Main themes identified through qualitative thematic analysis of open-ended responses from surveyed veterinarians in Málaga, Spain (2025 *n* = 47). Main themes identified through inductive thematic analysis of the 47 open-ended responses. Comments were independently coded by two researchers and grouped into overarching themes. Individual comments could contribute to more than one theme. The figure summarizes the principal facilitators, barriers, and suggested actions regarding occupational vaccination among veterinarians.

A total of 47 participants provided open-ended comments. Qualitative analysis identified three overarching domains: positive perceptions of vaccination, barriers to occupational immunization, and proposed actions to improve vaccine uptake (Figure 2).

Overall, respondents expressed highly positive attitudes toward vaccination. The most frequently identified theme was the recognition of vaccination as an essential measure to protect both veterinary professionals and public health (25/47 comments, 53.2%), particularly in relation to zoonotic diseases. In addition, 18 participants (38.3%) emphasized that vaccination should be considered an integral component of routine occupational health protection for veterinarians.

Participants also identified several barriers limiting vaccine uptake. The most frequently reported barrier was “limited access to recommended vaccines”, particularly rabies vaccine (24/47, 51.1%). Other commonly reported barriers included the “absence of structured occupational vaccination programmes” (20/47, 42.6%), “insufficient profession-specific information” (18/47, 38.3%), and “limited awareness among primary healthcare professionals regarding vaccination recommendations for veterinarians” (15/47, 31.9%). Concerns about adverse effects, mainly related to COVID-19 vaccination, were mentioned by 8 participants (17.0%), while 6 respondents (12.8%) suggested that vaccination recommendations should be better adapted according to individual occupational risk.

Finally, respondents proposed several practical measures to strengthen occupational immunization. The most frequent recommendations included the development of “profession-specific vaccination programmes” (22/47, 46.8%), the establishment of “official risk-based vaccination recommendations” (19/47, 40.4%), “improved access to vaccines through Veterinary Associations and public health services” (17/47, 36.2%), and “greater continuing education in vaccinology” (16/47, 34.0%). Overall, the qualitative findings indicate that veterinarians are highly supportive of vaccination but perceive important organizational barriers that hinder the implementation of effective occupational immunization strategies.

## 4. Discussion

### 4.1. Principal Findings

This study provides new evidence on self-reported vaccination coverage and vaccination perceptions among actively practicing veterinarians in Málaga, Spain. Although most respondents considered vaccination an important measure for personal and public health protection, self-reported coverage varied considerably across the vaccines evaluated. Uptake was particularly low for seasonal influenza and completion of the hepatitis B vaccination schedule, while COVID-19 vaccination coverage was high, likely reflecting the exceptional public health response during the pandemic. In addition, nearly half of the respondents were uncertain about their hepatitis B vaccination status, suggesting limitations in vaccination records, recall, or occupational immunization monitoring rather than confirmed lack of vaccination. This finding highlights the importance of maintaining accessible occupational vaccination records and facilitating verification of vaccination status throughout professional careers.

These findings should be interpreted in the context of vaccine-specific occupational recommendations. Not all vaccines assessed are indicated for every veterinarian, as recommendations vary according to professional activity, occupational exposure, travel, and individual risk factors. Nevertheless, the results suggest that, even for vaccines recommended for many veterinary professionals, vaccination uptake remains limited among surveyed veterinarians.

Overall, the coexistence of highly favourable attitudes toward vaccination and relatively low self-reported uptake for several recommended vaccines suggests that factors beyond vaccine acceptance may influence vaccination behaviour. Qualitative findings indicate that limited access to vaccines, insufficient visibility of occupational vaccination programmes, and unmet training needs may represent important barriers that deserve further investigation.

Taken together, these findings provide a descriptive overview of current vaccination practices among surveyed veterinarians and identify several areas where occupational vaccination policies, access to immunization, and professional guidance could be strengthened within a One Health framework.

### 4.2. Vaccination Coverage Among Veterinarians: Comparison with Existing Evidence

Vaccination coverage among the surveyed veterinarians differed substantially according to the vaccine considered and should be interpreted in light of the corresponding occupational recommendations. Whereas vaccines such as seasonal influenza are broadly recommended for veterinary professionals because of their potential exposure to animal and human influenza viruses, others—particularly rabies vaccination—are primarily indicated for veterinarians with specific occupational exposure or travel-related risk. Likewise, hepatitis B and Td/Tdap vaccination are largely based on routine adult immunization schedules, although occupational circumstances may reinforce their recommendation.

Seasonal influenza vaccination coverage (21.3%) was considerably lower than that reported among healthcare workers in Spain (approximately 39.5%) and remained far below the 75% coverage target recommended by the Spanish Ministry of Health, the World Health Organization, and European public health authorities for healthcare personnel [13,14]. Similar discrepancies between recommended targets and actual uptake have been reported across European countries, where influenza vaccination coverage among healthcare workers remains highly variable [14].

Published evidence specifically addressing vaccination coverage among veterinarians remains scarce, reflecting the limited surveillance of occupational immunization in this professional group. Available studies show substantial heterogeneity between countries, with relatively high rabies vaccination coverage reported where occupational vaccination programmes are well established, whereas lower uptake has been described for influenza, tetanus, Q fever, and other recommended vaccines in different veterinary settings [15,16]. Direct comparisons should nevertheless be interpreted cautiously because vaccination recommendations, epidemiological contexts, and occupational exposure profiles differ considerably across countries.

In Spain, recent national recommendations explicitly recognize veterinarians as a professional group for whom seasonal influenza vaccination should be considered because of potential exposure to avian and swine influenza viruses. Rabies pre-exposure vaccination is likewise recommended only for professionals whose occupational activities involve a significant risk of exposure [17,18]. Consequently, the relatively low overall rabies vaccination coverage observed in this survey should not be interpreted as inadequate for the veterinary profession as a whole but rather as an indicator warranting further assessment among veterinarians with specific occupational risk.

Taken together, these findings suggest that the implementation of occupational vaccination recommendations remains heterogeneous. Beyond the existence of formal recommendations, improving awareness, facilitating access to indicated vaccines, and strengthening occupational vaccination programmes may be necessary to increase vaccine uptake among veterinary professionals [19].

International veterinary and One Health organizations could play an important role by promoting harmonized evidence-based occupational vaccination recommendations, thereby reducing the current heterogeneity between countries and facilitating their implementation in routine veterinary practice.

### 4.3. Perceptions, Attitudes, and Barriers to Vaccination

Most surveyed veterinarians expressed highly favourable attitudes toward vaccination. More than 85% assigned high importance to vaccination as a measure for personal protection and public health, and qualitative responses consistently reflected positive perceptions of immunization. These findings are consistent with previous studies reporting generally favourable attitudes toward vaccination among both healthcare and veterinary professionals [15,20].

Despite these positive perceptions, self-reported vaccination coverage remained limited for several vaccines recommended for many veterinary professionals, particularly seasonal influenza and completion of the hepatitis B vaccination schedule. Although the present study did not directly assess vaccination intention or behavioural determinants, the coexistence of favourable attitudes and relatively low uptake suggests that factors other than vaccine acceptance may influence vaccination practices.

The qualitative findings provide useful insight into these factors. Respondents frequently identified limited access to vaccines, insufficient visibility of occupational vaccination programmes, and unmet training needs in vaccinology as barriers to vaccination. Some participants also questioned the indication for certain vaccines according to their professional activity, highlighting the importance of risk-based recommendations and clear occupational guidance.

Improving vaccination uptake among veterinary professionals therefore requires more than increasing awareness alone. Effective strategies should combine evidence-based education, easier access to recommended vaccines, structured occupational vaccination programmes, and clear professional recommendations adapted to different veterinary practice settings [21,22,23,24]. Such interventions are consistent with the One Health approach by strengthening the protection of veterinary professionals while contributing to the prevention of infectious diseases at the human–animal interface [19,25,26].

The findings suggest that, in this professional group, improving vaccine uptake may depend less on increasing vaccine acceptance than on reducing organizational and access-related barriers to recommended occupational immunization.

### 4.4. Occupational and Public Health Implications Within a One Health Framework

Veterinarians occupy a unique position at the human–animal–environment interface and play a central role in the surveillance, prevention, and control of zoonotic diseases. Consequently, maintaining adequate vaccination among veterinary professionals represents an important component of occupational health protection and may also contribute to broader public health preparedness within a One Health framework [27,28].

The present findings indicate that self-reported vaccination coverage for several recommended vaccines remains limited among surveyed veterinarians, although the occupational indication for some vaccines varies according to professional activity and individual exposure risk. These results therefore reinforce the importance of implementing risk-based occupational vaccination strategies tailored to the diversity of veterinary practice rather than adopting a uniform approach for the profession as a whole.

From a One Health perspective, improving occupational vaccination programmes may strengthen the resilience of the veterinary workforce and facilitate coordinated prevention of infectious diseases at the human–animal interface [29,30,31]. Although this study did not assess zoonotic transmission, occupational exposure, or outbreak preparedness directly, the findings support the public health relevance of promoting evidence-based vaccination policies for veterinary professionals as part of integrated One Health prevention strategies.

Such strategies should combine harmonized occupational vaccination recommendations, improved access to indicated vaccines, continuing education in vaccinology, and closer collaboration between veterinary organizations, occupational health services, and public health authorities. Strengthening these components would contribute to protecting veterinary professionals while reinforcing multidisciplinary collaboration for the prevention and control of infectious diseases.

Beyond protecting individual professionals, occupational vaccination should be regarded as part of the preparedness capacity of veterinary services within One Health systems, particularly in the context of emerging and re-emerging zoonotic threats.

### 4.5. Policy Implications

The present findings highlight the need to strengthen occupational vaccination strategies for veterinary professionals through coordinated action by public health authorities, occupational health services, veterinary associations, employers, and international veterinary organizations. Vaccination policies should incorporate risk-based occupational recommendations reflecting the diversity of veterinary practice and patterns of professional exposure.

Practical measures include occupational vaccination records, periodic reminders, facilitated access to recommended vaccines, and continuing education in vaccinology. In addition, the development of evidence-based vaccination recommendations by international veterinary and One Health organizations could promote greater harmonization of occupational immunization policies and support their implementation at the national level.

Although conducted in a single Spanish province, this study identifies potentially modifiable barriers to vaccination. Within a One Health perspective, strengthening occupational vaccination programmes may improve workforce protection and contribute to preparedness against zoonotic threats at the human–animal interface. These initiatives are also consistent with recent recommendations from the Federation of Veterinarians of Europe, which recognizes veterinarians as essential health professionals and advocates improved occupational protection, including access to vaccination programmes [9].

### 4.6. Strengths and Limitations

As with any survey-based cross-sectional study, several limitations should be considered when interpreting these findings. First, participation was voluntary, resulting in a convenience sample with a modest response rate (15.2% of eligible veterinarians), which may have introduced selection bias. Although post-stratification weighting by age and sex was applied to reduce demographic imbalances, residual non-response bias cannot be excluded. Second, vaccination status was self-reported and was not verified through vaccination records or serological testing. Consequently, uncertainty regarding hepatitis B vaccination status should be interpreted as lack of recall or documentation rather than evidence of non-immunity. Third, the questionnaire was specifically developed for this study. Although it was pilot-tested among 20 veterinarians and minor modifications were introduced to improve clarity, it did not undergo formal psychometric validation. Finally, the study was conducted in a single Spanish province, which may limit the generalizability of the findings to other veterinary populations.

Despite these limitations, the study provides original data on vaccination coverage and perceptions among veterinarians, an occupational group that remains underrepresented in vaccination research. The inclusion of both quantitative and qualitative data offers complementary insights into vaccination practices, perceived barriers, and educational needs. Furthermore, the study identifies practical opportunities to strengthen occupational vaccination programmes and contributes empirical evidence to support the development of evidence-based vaccination recommendations for veterinary professionals within a One Health framework.

### 4.7. Future Research and Policy Directions

Further research is needed to better understand vaccination practices and determinants among veterinary professionals across different practice settings and countries [16]. Larger multicentre studies using representative samples would help confirm the generalizability of these findings and identify occupation-specific vaccination needs according to professional exposure. Longitudinal studies should also evaluate the effectiveness of targeted educational interventions, facilitated vaccine access, and structured occupational vaccination programmes in improving vaccine uptake [28,29].

Future research should support the development of evidence-based occupational vaccination recommendations for veterinary professionals, including risk-based immunization strategies adapted to different areas of veterinary practice. Such evidence would contribute to strengthening occupational health policies and advancing One Health approaches to the prevention of vaccine-preventable diseases at the human–animal interface [24].

## 5. Conclusions

Vaccination coverage among the surveyed veterinarians in Málaga was suboptimal for several vaccines relevant to occupational health, particularly seasonal influenza and Td/Tdap boosters, despite participants reporting highly positive attitudes toward vaccination. Particular attention should be paid to hepatitis B vaccination, as nearly half of the participants were unable to report their vaccination status, suggesting important deficiencies in vaccination awareness, documentation, or record accessibility rather than confirmed lack of vaccination. Although COVID-19 vaccination uptake was high, reflecting the exceptional circumstances of the pandemic and intensive public health campaigns, important gaps remain in the implementation of routine occupational immunization.

Considering that veterinarians work at the human–animal interface and are routinely exposed to biological hazards, vaccination should be regarded as a core component of occupational health protection within a One Health framework. The findings of this study suggest that improving vaccine uptake requires not only greater awareness but also better access to recommended vaccines, structured occupational vaccination programmes, and stronger collaboration between veterinary organizations, employers, and public health services.

The results also support the development of evidence-based occupational vaccination recommendations tailored to veterinary practice. Greater leadership from national and international veterinary organizations, together with One Health institutions, would help harmonize recommendations, facilitate implementation, and strengthen the protection of veterinary professionals while contributing to preparedness against zoonotic threats.

## Figures and Tables

**Table 1:** Sociodemographic and professional characteristics of surveyed veterinarians (2025).

Characteristic	*n* (%)
Total participants	164 (15.2% of actively practicing veterinarians)
Age (years) Mean ± SD	45.6 ± 12.8
Sex	
Female	104 (63.4%)
Male	59 (36.0%)
Not specified	1 (0.6%)
Age groups (years)	
<30	26 (15.9%)
30–39	26 (15.9%)
40–49	48 (29.3%)
50–59	47 (28.7%)
≥60	17 (10.4%)
Work setting	
Urban	110 (67.5%)
Rural	27 (16.6%)
Mixed urban–rural	4 (2.5%)
Not specified/trainees	16 (9.8%)
Main professional activity	
Small animal clinical practice	108 (65.9%)
Public health	18 (11.0%)
Clinical practice/livestock production	9 (5.5%)
Animal health	4 (2.4%)
Food/environmental safety	4 (2.4%)
Other	15 (9.1%)

Footnote: Participation was voluntary; the sample slightly overrepresents younger veterinarians and women compared with the total registered population.

**Table 2:** Vaccination coverage against vaccine-preventable diseases among veterinarians (Málaga, Spain, 2025).

Vaccine	Vaccination Status	*n* (%)
Seasonal influenza (2024–2025)	Vaccinated	35 (21.3%)
	Not vaccinated	129 (78.7%)
Influenza vaccination (last 10 years)	Vaccinated every year	12 (7.4%)
	Vaccinated some years	62 (38.0%)
	Never vaccinated	86 (52.8%)
	Do not know	3 (1.8%)
COVID-19	≥3 doses (2021–2024)	103 (62.8%)
	1–2 doses	56 (34.1%)
	Not vaccinated	5 (3.0%)
Hepatitis B	Fully vaccinated (3 doses)	27 (16.5%)
	Vaccinated, schedule unknown	30 (18.3%)
	Not vaccinated	27 (16.5%)
	Do not know	80 (48.8%)
Td/Tdap (last 10 years)	≥1 dose	77 (47.2%)
	No booster dose	74 (45.4%)
	Do not know	12 (7.4%)
Rabies (occupational risk)	Vaccinated	38 (23.3%)
	Not vaccinated	123 (75.5%)
	Do not know	2 (1.2%)
Travel-related vaccines	Yes	33 (20.4%)
	No	129 (79.6%)

Footnote: Values are presented as unweighted frequencies and percentages based on self-reported vaccination history. Percentages were calculated using the number of respondents who provided a valid answer for each individual vaccine; therefore, denominators vary across vaccines because participants could omit individual questions. Hepatitis A vaccination was assessed only as part of travel-related vaccination because it is not routinely included in the Spanish adult immunization schedule. Td/Tdap: tetanus–diphtheria/tetanus–diphtheria–acellular pertussis.

## Data Availability

The datasets generated and analysed during the current study are available from the corresponding author upon reasonable request.

## Appendix A

Questionnaire to Assess the Vaccination Status of Licensed Veterinarians in the Province of Málaga

Dear Veterinarian,

We invite you to participate in this study, which aims to assess the vaccination status of licensed veterinarians in the province of Málaga regarding tetanus-diphtheria, influenza, COVID-19, and other vaccines. Your responses will be anonymous and used exclusively for research purposes.

The questionnaire is brief and will take no more than 5–10 min to complete. By clicking “Continue,” you confirm that you understand the purpose of the study and consent to participate voluntarily; to do so, you must read and accept the informed consent form.

Thank you very much for your cooperation.

Do you agree to participate in this study?
YES___ NO___

Section 1: Sociodemographic Data
1. What is your age in years? ○ ________ ○ No answer

2. Gender: ○ Male ○ Female ○ Others ○ No answer

Section 2: Employment Data

3. In which area of the province of Málaga do you primarily work? (Select one) ○ Rural ○ Urban ○ No answer

4. What is your main professional activity? (Select one) ○ Small animal practice ○ Farm animal practice/production ○ Public health veterinarian ○ Animal health veterinarian ○ Food safety/environmental health ○ Other ○ No answer

Section 3: Vaccination Status

5. Do you believe you have received the vaccines included in the childhood immunization schedule? ○ Yes, all of them ○ Yes, but I don’t know if I received all the vaccines or all the doses ○ No, I have not received these vaccines ○ I don’t know if I have received them or not ○ No answer

6. In the last 10 years, do you recall receiving one or more doses of the tetanus-diphtheria or tetanus-diphtheria-pertussis vaccine? ○ Yes, at least one dose ○ Yes, more than 1–2 doses ○ No doses in the last 10 years ○ I don’t know if I’ve received it or not ○ No answer

7. Did you receive the flu vaccine during the last flu season (2024–2025)? ○ Yes ○ No ○ I don’t know if I received it or not ○ No answer

8. Have you received the flu vaccine during any of the previous flu seasons in the last 10 years? ○ Yes, every year ○ Yes, some years ○ No ○ I don’t know if I’ve received it or not ○ No answer

9. Have you been vaccinated against COVID-19? ○ Yes, I received one or two doses during the 2021–2022 pandemic ○ Yes, I received more than two doses between 2021 and 2024 ○ No, I haven’t received any doses ○ I don’t know if I’ve been vaccinated or not ○ No answer

10. Do you think you have ever been vaccinated against hepatitis B? ○ Yes, I received the full 3-dose series as a child/teenager/adult ○ Yes, I think I’ve received some doses, but I don’t know if I have 3 doses ○ No, I haven’t received any doses ○ I don’t know if I’ve been vaccinated or not ○ No answer

11. Have you received any travel vaccines in the last 10 years? ○ Yes, the following: ___________________________ ○ Yes, although I don’t remember which ones ○ No, I haven’t received any doses for travel purposes ○ I don’t know if I’ve received them or not ○ No answer

12. Have you ever received vaccinations during your career as a veterinarian to protect against a specific risk, such as rabies or others? ○ Yes, I have been vaccinated against rabies ○ Yes, another vaccine ____________________________________ ○ No, I have not received any doses to protect against a specific risk of exposure as a veterinary professional ○ I do not know whether I have received it or not ○ No answer

Section 4: Additional Comments

13. What is your perception of the importance of vaccinations for veterinary professionals in terms of personal protection and global public health? Rate on a scale of 1 to 10, where 1 is “minimally important” and 10 is “very important”: ___________

14. Are there any comments you would like to add regarding your vaccination status or your opinion on vaccines? (Open-ended) ______________________________________________________________ ______________________________________________________________ ______________________________________________________________ ______________________________________________________________

Thank you for your participation.
